# Metabolomics Evaluation of Patients With Stage 5 Chronic Kidney Disease Before Dialysis, Maintenance Hemodialysis, and Peritoneal Dialysis

**DOI:** 10.3389/fphys.2020.630646

**Published:** 2021-01-20

**Authors:** Sang Zhu, Feng Zhang, Ai-Wen Shen, Bo Sun, Tian-Yi Xia, Wan-Sheng Chen, Xia Tao, Sheng-Qiang Yu

**Affiliations:** ^1^Department of Pharmacy, Changzheng Hospital, Naval Medical University, Shanghai, China; ^2^Department of Nephrology, Changzheng Hospital, Naval Medical University, Shanghai, China; ^3^Department of Pharmacy, The Affiliated Wuxi Children’s Hospital of Nanjing Medical University, Wuxi, China

**Keywords:** hemodialysis, peritoneal dialysis, predialysis CKD-5, metabolomics, UPLC-MS/MS

## Abstract

**Objective:**

Current treatment options for patients with stage 5 chronic kidney disease before dialysis (predialysis CKD-5) are determined by individual circumstances, economic factors, and the doctor’s advice. This study aimed to explore the plasma metabolic traits of patients with predialysis CKD-5 compared with maintenance hemodialysis (HD) and peritoneal dialysis (PD) patients, to learn more about the impact of the dialysis process on the blood environment.

**Methods:**

Our study enrolled 31 predialysis CKD-5 patients, 31 HD patients, and 30 PD patients. Metabolite profiling was performed using a targeted metabolomics platform by applying an ultra-high-performance liquid chromatography-tandem mass spectrometry method, and the subsequent comparisons among all three groups were made to explore metabolic alterations.

**Results:**

Cysteine metabolism was significantly altered between predialysis CKD-5 patients and both groups of dialysis patients. A disturbance in purine metabolism was the most extensively changed pathway identified between the HD and PD groups. A total of 20 discriminating metabolites with large fluctuations in plasma concentrations were screened from the group comparisons, including 2-keto-D-gluconic acid, kynurenic acid, s-adenosylhomocysteine, L-glutamine, adenosine, and nicotinamide.

**Conclusion:**

Our study provided a comprehensive metabolomics evaluation among predialysis CKD-5, HD, and PD patients, which described the disturbance of metabolic pathways, discriminating metabolites and their possible biological significances. The identification of specific metabolites related to dialysis therapy might provide insights for the management of advanced CKD stages and inform shared decision-making.

## Introduction

Chronic kidney disease (CKD) has received significant attention and has become a public health problem over recent decades. As renal function decreases during the course of disease progression in CKD patients, numerous uremic toxins gradually accumulate in the body, eventually causing symptoms of uremia, and various complications (Castner, 2010; Jürgensen et al., 2010; Zhang et al., 2012; Saran et al., 2015; Tonelli et al., 2016). Therefore, to maintain normal metabolic homeostasis, patients in stage 5 CKD are often required to undergo dialysis or prepare for transplantation programs. Although kidney transplantation is the preferred treatment method for patients with end-stage renal disease (ESRD), most patients are placed on dialysis while awaiting transplantation because of limited available donor kidneys (Fenton et al., 1997). Although hemodialysis (HD) is more used worldwide, the rate of peritoneal dialysis (PD) use has increased and is relatively more common among younger age groups (Saran et al., 2015). Similar outcomes have been reported for PD and HD in several recent studies, and the treatment options available for patients with stage 5 CKD before dialysis (predialysis CKD-5 patients) are primarily determined by individual circumstances, economic factors, and the doctor’s advice, although HD remains the most common initial modality for pediatric patients (Gilmore, 2006; Saran et al., 2015). In addition, studies comparing survival between these modalities have yielded conflicting results (Wang et al., 2020).

Dialysis is a complex procedure. Due to the continuous cleaning of toxins and the removal of excess water performed by dialysis, the metabolic status of the patient’s body becomes universally altered during the blood purification process. Dialysis efficiency is typically estimated by the clearance of urea, and other clinical indexes, including creatinine, hemogram parameters, and glucose levels. Thus, most studies capture only a very small portion of the information contained in the metabolome, such as nutritional or inflammatory indicators, a small number of special uremic toxins, as well as some mineral metabolism (Ilcol et al., 2002; Evans, 2003; Evenepoel et al., 2016). However, the metabolic changes that occur in the plasma after maintenance dialysis remains poorly understood; better characterizing these changes would provide profound information for the understanding of the traits and differences between PD and HD.

Currently, metabolomics offers a sophisticated and important platform for studying the biochemicals (or small molecules) present in cells, tissues, and body fluids (Chen et al., 2016). Metabolic alterations have been reported between HD patients and healthy volunteers (Rhee et al., 2010; Sato et al., 2011), between HD patients with glucose-added compared with glucose-free dialysate (Cui et al., 2013), and between HD patients with different metabolic phenotypes (Kalim et al., 2015; Liu et al., 2017). Only one report examined differences in serum metabolites between HD, PD, and normal healthy controls by using ^1^H-nuclear magnetic resonance (NMR)-based metabolomics, which indicated apparent differences in several metabolites linked to glucose metabolism and the tricarboxylic acid cycle between HD and PD patients (Choi et al., 2011). In addition, this study revealed that hypoxanthine and inosine were present only in patients receiving HD, which revealed increased levels of hypoxic and oxidative stress that might be attributed to HD. However, disturbances in metabolic pathways and disrupted relationships between metabolites and clinic data have not been mentioned in this NMR study.

Understanding the changes in serum metabolites that are associated with various dialysis modalities is important for facilitating information recovery according to dialysis treatments. In addition, the liquid chromatography (LC)/mass spectrometry (MS) method has recently become a dominant analytical tool, which has enabled major advances in global metabolite profiling compared with NMR spectroscopy, due to more robust and reproducible metabolic data, increased sensitivity and specificity for metabolite detection, and an increased linear dynamic range for metabolite determination (Spratlin et al., 2009; Theodoridis et al., 2012; Gika et al., 2014). To date, no study had examined the metabolic differences between HD and PD patients by LC/MS. Therefore, we describe the application of LC/MS-based metabolomics for the comparison of predialysis CKD-5, HD, and PD patients, for the first time. With this approach, we aimed to investigate the metabolic traits of patients in each group and their distributions along metabolic pathways, as well as latent relationships between metabolic alterations and clinical data.

## Materials and Methods

### Patients and Study Design

A cross-sectional cohort study of 61 stable uremia patients treated with dialysis for at least 6 months (HD, 31 patients; PD, 30 patients) and 31 predialysis CKD-5 patients without any history of dialysis therapy were enrolled in our study at Changzheng hospital (Shanghai, China). Subjects on HD were treated with three 4-h sessions each week during daytime. PD patients were treated with a total of 8–10 L of 1.5% with/without 2.5% low calcium peritoneal dialysate every day. All patients were aged 18 years or older and on a stable treatment schedule. We exclude patients with peritonitis, severe infections, initiation of PD for acute renal injury, a history of HD, or renal transplantation before PD and had no severe concomitant disease, or who had undergone a recent surgical procedure. The study was approved by the Ethics Committee of Changzheng Hospital, and informed consent was obtained from all patients.

Peripheral venous blood was collected from participants, and placed into a vacutainer containing EDTA-K2. All patients were sampled in the early morning on an empty stomach, at the same time, HD patients were predialysis blood on the day of HD. All PD patients underwent continuous ambulatory PD with stable blood biochemical indexes and internal environment before and after dialysis. Plasma samples were separated by centrifugation at 2,000 rpm for 20 min at 4°C and immediately frozen at −80°C for metabolomics analysis.

### Quantitation of 182 Metabolites

The 182 polar metabolites identified by our analysis were selected for their significant biological functions, including nucleotide metabolism, amino acid metabolism, carbohydrate metabolism, energy metabolism, the metabolism of cofactors and vitamins, glycan biosynthesis and metabolism, and lipid metabolism and choline metabolism (Supplementary Table 1).

All metabolites were detected using a published LC-MS/MS method, with slight modifications, to quantitate 200 targeted metabolites (Robinson et al., 2020). The modified, targeted, metabolomics platform was performed using a UHPLC system (1290 series, Agilent Technologies, United States) coupled to a triple quadrupole mass spectrometer (Agilent 6495 QQQ, Agilent Technologies, United States) in the multiple reaction monitoring (MRM) mode with positive/negative polarity switching. Chromatography separation was achieved on a Waters ACQUITY UPLC BEH Amide column (particle size, 1.7 μm; 100 mm × 2.1 mm), and the column temperature was maintained at 25°C. The flow rate was set to 300 μl/min, and the sample injection volume was 2 μl. The mobile phase A was 25 mM ammonium hydroxide and 25 mM ammonium acetate in 100% water, whereas mobile phase B was 100% acetonitrile, in both positive and negative mode. The linear gradient was set as follows: 0-1 min: 95% B, 1-14 min: 95% B to 65% B, 14-16 min: 65% B to 40% B, 16-18 min: 40% B, 18-18.1 min: 40% B to 95% B, and 18.1-23 min: 95% B. Electrospray ionization (ESI) source conditions were set as follows: gas temperature, 350°C; gas flow, 16 L/min; nebulizer, 40 psi; sheath gas temperature, 350°C; sheath gas flow, 12 L/min; capillary voltage, 3,000 V in positive mode or 2,500 V in negative mode; and nozzle voltage, 1,000 V in positive mode or 1,500 V in negative mode.

For sample preparation, 800 μl ACN:MeOH (1:1, v/v) was added to each 200 μl plasma sample to precipitate proteins. The supernatant was dried and dissolved in 100 μl of ACN:H_2_O (1:1, v/v) for LC-MS/MS analysis.

A quality control (QC) sample was prepared by mixing all biological samples and was run after every nine injections to evaluate the reproducibility of the instrument. A retention time quality control (RTQC) sample was prepared by mixing ten representative metabolites and was injected at the beginning, in the middle, and at the end of each batch to ensure the stability of retention time.

Targeted metabolomics data processing was performed in the MRM Analyzer package, including “pseudo” accurate *m*/*z* transformation, peak detection and alignment, metabolite identification, QC check, and statistical analysis.

### Statistical Analysis

Each patient’s demographic parameters and laboratory results were expressed as the mean ± standard deviation (SD) or the median (interquartile range), as appropriate. One-way analysis of variance (ANOVA) or the Kruskal–Wallis rank-sum test was performed to compare data among the three groups. Differences between two groups were evaluated by the least significant difference (LSD) test or Dunnett’s T3 test. The results were considered significant when *p* < 0.05. The SMICA13.0 software was used to perform multivariate statistical analysis. The identification of differential metabolites was performed by orthogonal projection to latent structure discriminant analysis (OPLS-DA). Additionally, the Kruskal–Wallis *H* test was used to determine the significance of metabolites within each comparison group. Pathway analysis and topology maps were performed by MetaboAnalyst^1^. Relationships between discriminating metabolites and clinical data were calculated using Pearson’s correlation coefficient. Heatmaps were generated using MeV version 4.9.0.

## Results

### Patient Characteristics

Demographic and clinical data are shown in Table 1. No significant differences in age and body mass index were identified among the patient groups. The percentage of males was higher than that of females for the PD and predialysis CKD-5 groups. All patients were treated with regular drug therapy to prevent anemia and hyperphosphatemia. Although plasma albumin and hemoglobin levels remained below normal levels in all three groups, the phosphorus level remained above the normal range. HD patients also received the additional administration of L-carnitine. Other clinical data were within the normal range in all three groups, except blood urea nitrogen (BUN) and creatinine.

**TABLE 1:** Demographic and clinical characteristics of patients.

	HD	PD	Predialysis CKD-5	*p* value
*n*	31	30	31	
Age	56.4 ± 10.6	51.4 ± 13.4	53.0(32.0,60.0)	0.112
Male/female	13/18	23/7	22/9	0.010
Body weight (kg)	63.1 ± 8.7	67.0 ± 9.0	63.7 ± 9.2	0.203
BMI (Kg/m^2^)	23.4 ± 2.4	23.9 ± 2.9	22.4 ± 2.4	0.086
Dialysis duration (months)	48.0(12.0,84.0)	27.0(12.0,48.0)	−	0.081
**Renal diagnosis, *n* (%)**				
Diabetic nephropathy	5(16.1)	4(13.3)	4(12.9)	
Glomerulonephritis	9(29.0)	20(66.7)	17(54.8)	
Hypertensive/large vessel disease	2(6.5)	3(10.0)	2(6.5)	
Cystic/hereditary/congenital disease	6(19.4)	0(0)	4(12.9)	
Postrenal transplantation/After nephrectomy	5(16.1)	0(0)	0(0)	
Unknown	4(12.9)	3(10.0)	4(12.9)	
BUN	19.0(15.6,23.5)^a^	21.3 ± 7.7	24.8(18.0,29.4)	0.032
Uric acid	342.9 ± 96.4^a,b^	410.9 ± 125.5	463.5 ± 125.0	< 0.001
Creatinine	884.2 ± 248.6^a,b^	1086.3 ± 294.8^a^	686.5 ± 175.3	< 0.001
Glucose	5.0(4.2,6.9)	4.6(4.1,5.4)	4.6(4.2,5.3)	0.378
AKP	108.9 ± 47.6	76.5(64.5,98.0)	83.8 ± 25.2	0.059
Albumin (g/dL)	38.8 ± 4.1^a,b^	30.5 ± 4.8^a^	34.4 ± 5.1	< 0.001
Hemoglobin	97.7 ± 18.9^a^	94.0 ± 18.6^a^	84.1 ± 18.0	0.014
Calcium	2.4 ± 0.3^a^	2.3(2.1,2.5)^a^	2.1 ± 0.3	< 0.001
Phosphorus	2.0 ± 0.5	1.9 ± 0.7	1.8(1.6,2.1)	0.529
Sodium	138.5 ± 3.8	139.5(136.0,142.3)	138.0 ± 5.3	0.616
Chlorine	99.4 ± 3.3^a^	98.6 ± 4.7^a^	102.5 ± 4.7	0.001
Potassium	4.6 ± 0.7^b^	3.7 ± 0.9^a^	4.3 ± 0.7	< 0.001
HCO_3_^–^	21.0(19.0,24.0)^b^	25.3 ± 4.5^a^	20.0 ± 4.9	< 0.001
PTH	415.1 ± 395.4	335.4 ± 271.5	248.6 ± 210.4	0.380
CRP	16.2 ± 28.4^a^	33.9 ± 48.9^a^	5.6 ± 5.5	0.005

*HD, hemodialysis; PD, peritoneal dialysis; BMI, body mass index; BUN, blood urea nitrogen; PTH, parathyroid hormone; AKP, Alkaline phosphatase; and CRP, C-reactive protein. ^*a*^HD or PD patients compared to predialysis CKD-5 patients, *p* < 0.05. ^*b*^HD patients compared with PD patients, *p* < 0.05.*

Compared with predialysis CKD-5 patients, HD patients had higher levels of creatinine, albumin, hemoglobin, and calcium, but lower levels of BUN, uric acid, and chlorine. PD patients had higher levels of creatinine, hemoglobin, calcium, and HCO_3_^–^, but lower levels of potassium, chlorine, and albumin compared with predialysis CKD-5 patients. Several parameters, including uric acid, creatinine, albumin, potassium, and HCO_3_^–^, showed significant differences between HD and PD patients.

### Metabolic Profiles Among Predialysis CKD-5, HD, and PD Groups

Typical total ion chromatograms for 3 representative plasma samples, from each of the predialysis CKD-5, HD, and PD groups, are shown in Supplementary Figure 1. A total of 83 metabolite peaks were identified by significantly changed levels among the three groups. To characterize specific metabolites and metabolic pathways for each group, first, differences in the levels of plasma metabolites between HD and predialysis CKD-5 groups and between PD and predialysis CKD-5 groups were analyzed to elucidate the influence of longtime dialysis procedures and dialysis type compared with CKD-5, and then differences in the levels of plasma metabolites were examined between HD and PD were assessed to investigate the differences in metabolic status between HD and PD.

**FIGURE 1:**
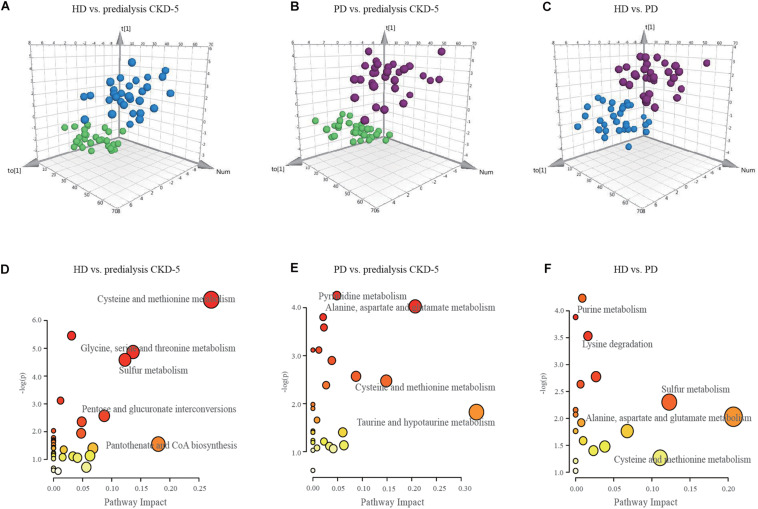
OPLS-DA score plots and disturbed metabolic pathways. The orthogonal projection to latent structure-discriminant analysis (OPLS-DA) score plots compared **(A)** HD to predialysis CKD-5, **(B)** PD to predialysis CKD-5, and **(C)** HD to PD. The disturbed metabolic pathways showed various metabolism changes when comparing **(D)** HD to predialysis CKD-5, **(E)** PD to predialysis CKD-5, and **(F)** HD to PD.

As shown in Figures 1A–C, OPLS-DA analysis exhibited discrimination among the predialysis CKD-5, HD, and PD groups: HD vs. predialysis CKD-5, R2Y at 0.853 and Q2 at 0.654; PD vs. predialysis CKD-5, R2Y at 0.7 and Q2 at 0.5; and HD vs. PD, R2Y at 0.7 and Q2 at 0.5. Each model achieved excellent separation, with good fitting and predictive capabilities. Subsequent selected important metabolites were determined by VIP (variable importance in projection)-values (>1.0) and *p*-values (<0.05). There were 27, 27, and 15 metabolites that satisfied the criteria for HD vs. predialysis CKD-5, PD vs. predialysis CKD-5, and HD vs. PD, respectively. All metabolites were considered significant variables between groups and were examined for further pathway analyses. An overview of these variables (a total of 42) for each comparison between groups can be found in Supplementary Table 2.

Compared with predialysis CKD-5 patients, 15 metabolites were elevated in both HD and PD patients, including kynurenic acid (HD vs. predialysis CKD-5, FC = 3.85; PD vs. predialysis CKD-5, FC = 4.70), purine (HD vs. predialysis CKD-5, FC = 3.80; PD vs. predialysis CKD-5, FC = 6.22), 2-keto-D-gluconic acid (HD vs. predialysis CKD-5, FC = 1.50; PD vs. predialysis CKD-5, FC = 3.72), argininosuccinic acid (HD vs. predialysis CKD-5, FC = 2.03; PD vs. predialysis CKD-5, FC = 1.63), *S*-adenosylhomocysteine (HD vs. predialysis CKD-5, FC = 1.79; PD vs. predialysis CKD-5, FC = 1.51), and 9 other metabolites. Specifically, the plasma level of L-carnitine (FC = 3.10) only increased in HD patients, whereas nicotinamide (FC = 1.37) levels only increased in PD patients. Glycerophosphocholine (FC = 1.65) and L-homocysteine (FC = 1.41) levels only decreased in HD patients, whereas L-glutamine (FC = 1.18) and L-leucine (FC = 1.13) levels only decreased in PD patients.

Comparing HD and PD patients, L-carnitine, adenosine, L-2-aminoadipic acid, L-glutamine, and flavone were higher in HD patients than in PD patients. In contrast, 10 metabolites, including 2-keto-D-gluconic acid, citraconic acid, adenosine, betaine, and nicotinamide, were higher in PD patients than in HD patients.

### Perturbed Pathways Identified in Group Comparisons

Because 42 metabolite concentrations showed significant changes in plasma concentrations among the three groups, pairwise comparisons were performed to determine which pathways were perturbed under different conditions. As shown in Figures 1D–F, the topology maps presented perturbed pathways for the three groups based on MetaboAnalyst: (1) cysteine-methionine metabolism, glycine-serine-threonine metabolism, sulfur metabolism, and pantothenate-CoA biosynthesis for HD vs. predialysis CKD-5; (2) taurine-hypotaurine metabolism, alanine-aspartate-glutamate metabolism, and cysteine-methionine metabolism for PD vs. predialysis CKD-5; and (3) alanine-aspartate-glutamate metabolism, cysteine-methionine metabolism, sulfur metabolism, and purine metabolism for HD vs. PD.

Cysteine metabolism appeared to be altered in all groups, including the dysregulation of L-serine, L-homocysteine, *S*-adenosylhomocysteine, and 5′-methylthioadenosine. Changes in purine metabolism were the most significant in the comparison between HD and PD. Higher levels of L-glutamine and adenosine and lower level of xanthosine in the plasma were found in HD patients compared with PD patients.

### Association Between Main Discriminating Metabolites and Clinical Data

**FIGURE 2:**
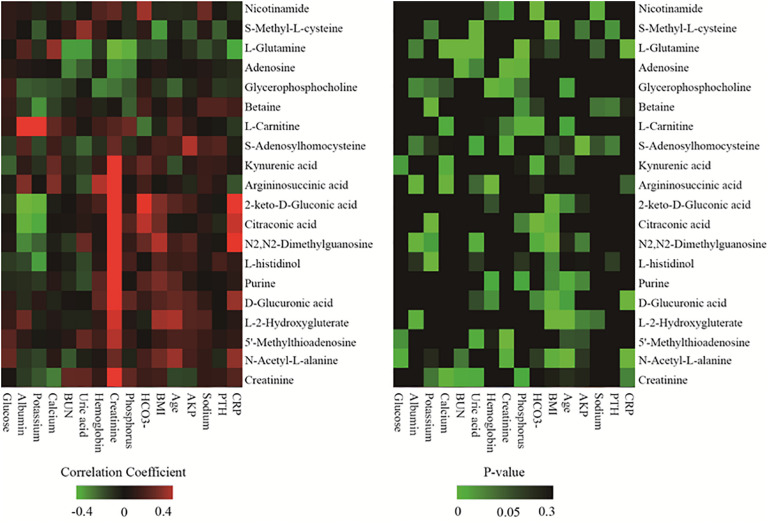
Heatmap of correlation analysis between 20 discriminating metabolites and various clinical parameters.

To further determine the relationship between the identified differential metabolites and clinical data, the discriminating metabolites were listed only when they met the following criteria: VIP > 1.5 and *p* < 0.01. A total of 20 of 83 metabolites met these criteria and are listed in Table 2. The selected metabolites had greater concentration fluctuations for each pairwise comparison. Correlations between the levels of these 20 discriminating metabolites and various clinical parameters are shown in Figure 2. Among the 20 discriminating metabolites, the levels of 11 metabolites had strong positive correlations with creatinine levels. 2-keto-D-Gluconic acid, citraconic acid, N2,N2-dimethylguanosine, D-glucuronic acid and *N*-acetyl-L-alanine were positively correlated with CRP, and L-glutamine negatively correlated with CRP levels. Calcium levels were positively correlated with argininosuccinic acid and L-glutamine. BUN and phosphorus levels were both negatively correlated with L-glutamine and adenosine levels. Hemoglobin levels were positively correlated with argininosuccinic acid levels and negatively correlated with glycerophosphocholine levels. Albumin levels were positively correlated with argininosuccinic acid, L-carnitine levels, and negatively correlated with 2-keto-D-gluconic acid, citraconic acid, and N2,N2-dimethylguanosine levels.

**TABLE 2:** Statistical analysis of 20 main discriminating metabolites.

	Metabolites	KEGG	HMDL	VIP	*p*	FC	Direction	RSD%
HD vs. CKD5	Kynurenic acid	C01717	HMDB00715	2.89	< 0.001	3.85	Up	13.89
	*L*-2-Hydroxygluterate	C03196	HMDB00694	2.19	< 0.001	2.71	Up	18.22
	5′-Methylthioadenosine	C00170	HMDB01173	1.90	< 0.001	1.82	Up	25.55
	N2,N2-Dimethylguanosine	NA	HMDB04824	1.90	< 0.001	1.77	Up	8.22
	L-Carnitine	C00318	HMDB00062	1.88	< 0.001	3.10	Up	6.97
	D-Glucuronic acid	C00191	HMDB00127	1.85	< 0.001	1.57	Up	8.78
	*S*-Adenosylhomocysteine	C00021	HMDB00939	1.77	< 0.001	1.79	Up	11.07
	Argininosuccinic acid	C03406	HMDB00052	1.67	0.001	2.03	Up	7.94
	*N*-Acetyl-L-alanine	NA	HMDB00766	1.66	0.004	1.27	Up	10.47
	2-keto-D-Gluconic acid	C06473	NA	1.63	0.003	1.50	Up	12.42
	Purine	C15587	HMDB01366	1.61	< 0.001	3.79	Up	23.27
	Glycerophosphocholine	C00670	HMDB00086	1.57	< 0.001	1.65	Down	11.29
	*S*-Methyl-L-cysteine	NA	HMDB02108	1.55	0.001	1.56	Down	9.09
PD vs. CKD5	2-keto-D-Gluconic acid	C06473	NA	2.80	< 0.001	3.72	Up	12.42
	Citraconic acid	C02226	HMDB00634	2.75	< 0.001	3.03	Up	15.69
	*S*-Adenosylhomocysteine	C00021	HMDB00939	2.05	< 0.001	1.51	Up	11.07
	D-Glucuronic acid	C00191	HMDB00127	2.03	< 0.001	1.63	Up	8.78
	N2,N2-Dimethylguanosine	NA	HMDB04824	1.98	< 0.001	2.47	Up	8.22
	Kynurenic acid	C01717	HMDB00715	1.90	< 0.001	4.70	Up	13.89
	L-2-Hydroxygluterate	C03196	HMDB00694	1.78	< 0.001	2.03	Up	18.22
	Purine	C15587	HMDB01366	1.73	< 0.001	6.22	Up	23.27
	L-Histidinol	C00860	HMDB03431	1.58	< 0.001	2.00	Up	27.47
	L-Glutamine	C00064	HMDB00641	1.51	0.001	1.18	Down	5.28
HD vs. PD	2-keto-D-Gluconic acid	C06473	NA	3.42	< 0.001	2.48	Down	12.42
	Citraconic acid	C02226	HMDB00634	2.98	< 0.001	2.19	Down	15.69
	L-Carnitine	C00318	HMDB00062	2.57	< 0.001	4.17	Up	6.97
	Adenosine	C00212	HMDB00050	1.83	0.003	1.28	Up	32.25
	Creatinine	C00791	HMDB00562	1.76	0.002	1.10	Down	2.38
	Betaine	C00719	HMDB00043	1.71	0.017	1.25	Down	7.55
	*S*-Methyl-L-cysteine	NA	HMDB02108	1.68	0.003	1.43	Down	9.09
	Nicotinamide	C00153	HMDB01406	1.59	0.012	1.32	Down	7.35

**FIGURE 3:**
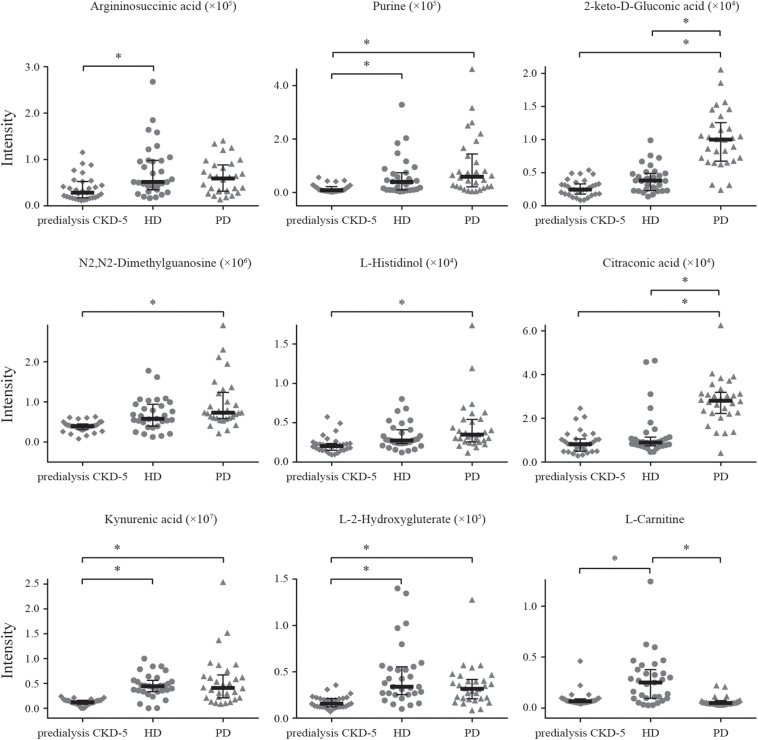
Alteration patterns of discriminating metabolites with fold-change > 2. * means the metabolite had large alteration between two groups, with fold-changes > 2.

Detection remained stable during analyses of the whole sample sequence, with the relative standard deviation (RSD) for the 20 discriminating metabolites varying from 2.4 to 32.3%. The fold-change varied from 1.1 to 6.2, which indicated enormous alterations in the levels of discriminating metabolites. Among the 20 discriminating metabolites, nine of them had large alterations, with fold-changes >2, as shown in Figure 3.

## Discussion

The selection of dialysis modality may be biased by many aspects, and a previous study reported a lack of simple answers to the question of which dialysis modality can be expected to provide a better quality of life. Several studies have suggested advantages for PD in some domains, whereas others have indicated the advantages of HD or reported minimal differences between the two modalities (Zhang et al., 2007; Hsu et al., 2020). PD requires a long-term peritoneal catheter, whereas HD requires vascular access, such as an arteriovenous fistula/graft or a venous catheter. The key achieving successful PD or HD was the management of the general clinical state of the patient, including good nutritional status and the prevention of anemia, edema, hypertension, electrolyte, and acid-base disturbances, neurologic symptoms, pruritus, and insomnia, which could be manifested by small molecules that are the intermediates and products of metabolism (Rippe, 2010).

To understand the impacts of HD or PD on the metabolites produced by predialysis CKD-5 patients, our study provided a comprehensive metabolomics evaluation, based on the ultra-high-performance liquid chromatography-tandem mass spectrometry (UHPLC-MS/MS) technique, to compare predialysis CKD-5 and maintenance HD and PD patients. This targeted metabolomics analysis had been demonstrated to be capable of identifying dysregulated metabolites and related metabolic pathways (Theodoridis et al., 2012). In this study, the plasma levels of 42 metabolites demonstrated significant differences among the three groups, most of which were involved in oxidative stress. In addition, cysteine-methionine metabolism was found to be the most significantly disturbed metabolism pathways in all pairwise comparisons, which indicated the occurrence of universal metabolic alterations due to the unselective clearance of plasma uremic toxins during dialysis. Although HD and PD were recognized as the most frequently prescribed methods for predialysis CKD-5 patients to remove uremic retention solutes from blood circulation, approximately two-thirds of the differential metabolites showed lower plasma levels in HD patients compared with PD patients, which suggested that the clearance of small molecules was better when using HD than PD, as previously reported (Evenepoel et al., 2016).

Among the 20 identified discriminating metabolites, argininosuccinic acid, purine, 2-keto-D-gluconic acid, N_2_,N_2_-dimethylguanosine, L-histidinol, kynurenic acid, *L*-2-hydroxyglutaric acid, *S*-adenosylhomocysteine, *N*-acetylglutamine, *N*-acetyl-*L*-alanine, 5′-methylthioadenosine, and D-glucuronic acid were found to be significantly increased in both HD and PD patients compared with predialysis CKD-5 patients (Table 2 and Figure 3), with significantly positive correlations observed between the levels of these metabolites and creatinine levels (Figure 2). For example, 2-keto-D-gluconic acid, a metabolite produced by bacteria, such as *Pseudomonas aeruginosa* and *Serratia marcescens*, was increased in both HD and PD patients compared with predialysis CKD-5 patients. Both exit-site/tunnel infections, such as infections associated with the venous catheter in PD patients and the peritoneal catheter in HD patients, could lead to the increased relative abundance of specific bacteria in the blood microbiome of HD patients and the increased relative abundance of other bacteria in the peritoneal microbiome of PD patients (Simões-Silva et al., 2018). This finding supports the observation that CRP showed significant rise in both PD (*P* < 0.01) and HD groups (*P* < 0.05). Besides, the abovementioned bacterial species are both Gram-negative organisms that can be found in PD patients and are likely to promote severe infections associated with poor outcomes (Misenheimer et al., 1965; Chia et al., 2008). Although 2-keto-D-gluconic acid levels were lower in HD patients than in PD patients, CRP values showed no differences between the two groups.

**FIGURE 4:**
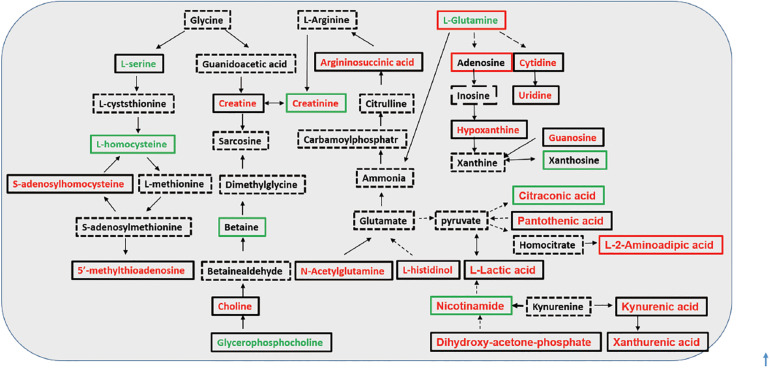
Investigated metabolic pathways for discriminating metabolites. Font colors (red and green) represent an up- or down-regulated metabolite level with significant changes in HD group, in comparison with that in the predialysis CKD-5 group; frame colors (red and green) represent an increase or decrease in metabolite level in HD group compared with PD group. Direct conversion between two metabolites was illustrated in full line, while indirect was in dash line.

During the last two decades, oxidative stress has emerged as a novel risk factor for accelerated atherosclerosis and elevated mortality in CKD and dialysis patients (Roumeliotis et al., 2019). In addition, the oxidative stress appears to be exacerbated by dialysis procedures, with HD patients presenting more enhanced oxidative stress than PD patients (Liakopoulos et al., 2017a,b). It was elucidated by some metabolites recognized as being pro- and antioxidants were found in different levels between the tested groups (Supplementary Table 3). Then, we depicted the investigated metabolic pathways associated with the identified discriminating metabolites among the three groups (Figure 4). Compared with predialysis CKD-5 patients, both HD and PD patients presented the upregulation of kynurenic acid and *S*-adenosylhomocysteine (SAH). Kynurenic acid, a well-known uremic toxin, was found to be retained in patients with CKD compared with healthy controls by metabolomics studies, and its elevated levels could activate the aryl hydrocarbon receptor pathway to increase tissue factor levels in the vessel wall to enhance thrombosis (Kolachalama et al., 2018; Roshanravan et al., 2018). The upregulation of SAH, the metabolic precursor of homocysteine, was shown to be associated with cardiovascular disease and increased mortality in predialysis CKD-5 patients (Rhee et al., 2010; Kalim et al., 2015; Liu et al., 2017). Studies have reported a high level of plasma SAH and whole-body transmethylation inhibition in HD patients (Choi et al., 2011), confirming our findings. L-glutamine, another substance related to cardiovascular function, presented lower levels in PD patients than in predialysis CKD-5 patients, and L-glutamine levels were significantly correlated with calcium levels (*r* = 0.241, *p* = 0.020) in our study. L-glutamine supplementation could improve vascular function before the development of uremia (Spratlin et al., 2009). The upregulation of kynurenic acid and SAH and the downregulation of L-glutamine observed in dialysis patients indicated an increased cardiovascular risk for both HD and PD patients compared with predialysis CKD-5 patients. In addition, D-glucuronic acid was increased in both HD and PD patients compared with predialysis CKD-5 patients. The significance of glucuronic acid during the detoxication process is well-known and favors the detoxication of foreign compounds within the body (Lundgren and DePierre, 1990).

As shown in Table 2, glycerophosphocholine was decreased and L-carnitine was increased in HD patients and argininosuccinic acid, purine, and N_2_,N_2_-dimethylguanosine were increased in both HD and PD patients compared with predialysis CKD-5 patients. High level of N2,N2-dimethylguanosine was associated with CKD cause, and glycerophosphocholine were found reduced in the maintenance HD patients when compared with healthy controls (Grams et al., 2017; Chen et al., 2020). Compared with PD patients, L-carnitine and adenosine were increased in HD patients, whereas nicotinamide, betaine, 2-keto-D-Gluconic acid, citraconic acid, creatinine, and *S*-Methyl-L-cysteine were reduced in HD patients. Nicotinamide was found decreased in the CKD patients when compared with the healthy controls (Roshanravan et al., 2018). Besides, it was proven to effectively reduce phosphorus absorption in the gastrointestinal tract, and a nicotinamide supplement has previously been used as a therapeutic intervention to reduce serum phosphorus (Shahbazian et al., 2011; El Borolossy et al., 2016). Carnitine depletion was shown to occur in uremia patients and was magnified by dialysis, which was associated with multiple adverse outcomes, including anemia and muscle weakness (Bellinghieri et al., 2003; Kalim et al., 2013). Therefore, in our study, the regular injection of carnitine supplements counteracted the loss of L-carnitine to maintain acylcarnitine homeostasis.

However, our study has several limitations. First, the sample size of this study was limited, and the gender distribution among there groups were slightly unbalanced. Therefore, stringent *p*-value thresholds were applied throughout. Furthermore, all metabolites were found to be significant relative to an age- and BMI-matched groups. A second limitation was that all subjects were recruited from a single research center. In addition, data should be obtained from patients over time to determine whether plasma-targeted metabolite profiling can offer information regarding the survival rate. Finally, the metabolite profiling in our study was performed using a targeted metabolomics method that analyzed 183 pre-specified metabolites; however, targeted metabolomics selectively measured a set of known essential metabolites in biologically relevant metabolic pathways, rather than identifying all metabolic changes. Some other key metabolites, such as folic acid, could not be assayed in this study, which is a limitation of this analysis method. Besides, as the metabolites were proved to provide reliable evidence of pathological process and the treatment outcome, it was meaningful for us to assess long-term longitudinal changes for each CKD 5 patients at baseline and after 1 year or longer remained on conventional PD or HD.

In conclusion, although the present study was not a randomized, controlled study, our study indicated that a metabolomics platform based on a UHPLC-MS/MS could be used as a discovery tool to analyze the metabolic traits of predialysis CKD-5, HD, and PD patients. Quantitative data revealed that 20 metabolites having correlation with clinical characteristics showed significant differences among these three groups. Compared with predialysis CKD-5 patients, upregulated kynurenic acid and SAH and downregulated L-glutamine levels were observed in dialysis patients, which indicated that cardiovascular risk assessments should be performed in uremia patients when they started dialysis. High level of 2-keto-D-gluconic acid accompanied with increased CRP indicated the high rates of infectious complications for both HD and PD patients. PD was recommended for predialysis CKD-5 patients with good peritoneal function to maintain cardiovascular stability.

## Data Availability Statement

The original contributions presented in the study are included in the article/Supplementary Material, further inquiries can be directed to the corresponding author/s.

## Ethics Statement

The studies involving human participants were reviewed and approved by Ethics Committee of Changzheng hospital. The patients/participants provided their written informed consent to participate in this study.

## Author Contributions

S-QY, XT, and W-SC designed and conducted the research. SZ, FZ, and A-WS performed the experiments and wrote the manuscript. BS and T-YX collected and analyzed the data. All authors contributed to the article and approved the submitted version.

## Conflict of Interest

The authors declare that the research was conducted in the absence of any commercial or financial relationships that could be construed as a potential conflict of interest.

## Footnotes

1 http://www.metaboanalyst.ca
