# Supplementary material for: Metabolomics Evaluation of Patients With Stage 5 Chronic Kidney Disease Before Dialysis, Maintenance Hemodialysis, and Peritoneal Dialysis
Source: Front Physiol. 2021 Jan 20;11:630646. doi: 10.3389/fphys.2020.630646 (PMC7855177; doi:10.3389/fphys.2020.630646)

**Appendix A. Supplementary material**

**Table S1.** Summary of global metabolomic analysis of 182 polar metabolites.

| Metabolic Pathway | **Total** | **Hits** |
| --- | --- | --- |
| **Nucleotide metabolism** |  |  |
| Purine metabolism | 45 | 21 |
| Pyrimidine metabolism | 36 | 17 |
| **Amino acid metabolism** |  |  |
| Glutathione metabolism | 10 | 6 |
| Methionine metabolism | 24 | 10 |
| Glutamate metabolism | 18 | 8 |
| Aspartate metabolism | 12 | 6 |
| Arginine and proline metabolism | 26 | 8 |
| Glycine, serine, and threonine metabolism | 26 | 7 |
| Alanine metabolism | 6 | 2 |
| Histidine metabolism | 11 | 3 |
| Tryptophan metabolism | 34 | 6 |
| Ornithine cycle | 20 | 10 |
| Alanine metabolism | 12 | 6 |
| Other ammonia recycling | 18 | 10 |
| **Carbohydrate metabolism** |  |  |
| Citric acid cycle | 23 | 10 |
| Inositol metabolism | 19 | 3 |
| Galactose metabolism | 25 | 4 |
| Glycolysis | 21 | 7 |
| Gluconeogenesis | 27 | 9 |
| Other carbohydrate metabolism | 19 | 4 |
| **Energy metabolism** |  |  |
| Oxidative phosphorylation | 15 | 7 |
| Malate-Aspartate shuttle | 8 | 5 |
| Glycerol phosphate shuttle | 8 | 4 |
| **Metabolism of cofactors and vitamins** |  |  |
| Thiamine metabolism | 4 | 3 |
| Nicotinate and nicotinamide metabolism | 13 | 6 |
| Biotin metabolism | 4 | 2 |
| **Glycan biosynthesis and metabolism** |  |  |
| Nucleotide sugars metabolism | 9 | 3 |
| **Lipid metabolism** |  |  |
| Phospholipid biosynthesis | 19 | 5 |
| Synthesis and degradation of ketone bodies | 10 | 3 |
| Glycerolipid metabolism | 13 | 3 |
| **Choline metabolism** | 10 | 5 |

**Table S2. Statistical analysis of 42 differential metabolites from each comparison groups**

|  | **Metabolites** | **KEGG** | **HMDL** | **RT (min)** | **VIP** | **p** | **Fold change** | **Trend** |
| --- | --- | --- | --- | --- | --- | --- | --- | --- |
| HD vs. | Kynurenic acid | C01717 | HMDB00715 | 5.749 | 2.89 | < 0.001 | 3.85 | up |
| predialysis CKD-5 | L-2-Hydroxygluterate | C03196 | HMDB00694 | 12.047 | 2.19 | < 0.001 | 2.71 | up |
|  | 5'-Methylthioadenosine | C00170 | HMDB01173 | 2.213 | 1.90 | < 0.001 | 1.82 | up |
|  | N2,N2-Dimethylguanosine | NA | HMDB04824 | 5.587 | 1.90 | < 0.001 | 1.77 | up |
|  | L-Carnitine | C00318 | HMDB00062 | 10.763 | 1.88 | < 0.001 | 3.10 | up |
|  | D-Glucuronic acid | C00191 | HMDB00127 | 11.864 | 1.85 | < 0.001 | 1.57 | up |
|  | S-Adenosylhomocysteine | C00021 | HMDB00939 | 11.718 | 1.77 | < 0.001 | 1.79 | up |
|  | Argininosuccinic acid | C03406 | HMDB00052 | 14.533 | 1.67 | 0.001 | 2.03 | up |
|  | N-Acetyl-L-alanine | NA | HMDB00766 | 7.568 | 1.66 | 0.004 | 1.27 | up |
|  | 2-keto-D-Gluconic acid | C06473 | NA | 11.226 | 1.63 | 0.003 | 1.50 | up |
|  | Purine | C15587 | HMDB01366 | 3.575 | 1.61 | < 0.001 | 3.79 | up |
|  | Glycerophosphocholine | C00670 | HMDB00086 | 11.936 | 1.57 | < 0.001 | 1.65 | down |
|  | S-Methyl-L-cysteine | NA | HMDB02108 | 8.723 | 1.55 | 0.001 | 1.56 | down |
|  | L-histidinol | C00860 | HMDB03431 | 10.268 | 1.46 | 0.001 | 1.46 | up |
|  | Pantothenic acid | C00864 | HMDB00210 | 8.117 | 1.42 | 0.001 | 1.39 | up |
|  | Uridine | C00299 | HMDB00296 | 4.631 | 1.37 | 0.024 | 1.25 | up |
|  | N-Acetylglutamine | NA | HMDB06029 | 9.397 | 1.35 | 0.005 | 1.64 | up |
|  | FAD | C00016 | HMDB01248 | 12.250 | 1.32 | 0.02 | 1.19 | down |
|  | Creatine | C00300 | HMDB00562 | 10.548 | 1.26 | 0.014 | 1.38 | up |
|  | Dihydroxy-acetone-phosphate | C00111 | HMDB01473 | 13.411 | 1.23 | 0.008 | 1.75 | up |
|  | L-Homocysteine | C00155 | HMDB00742 | 8.725 | 1.23 | 0.011 | 1.41 | down |
|  | L-Serine | C00065 | HMDB00187 | 11.450 | 1.16 | 0.026 | 1.16 | down |
|  | Hypoxanthine | C00262 | HMDB00157 | 5.011 | 1.14 | 0.012 | 1.58 | up |
|  | Flavone | C15608 | HMDB03075 | 1.097 | 1.11 | 0.018 | 1.39 | up |
|  | L-2-Aminoadipic acid | C00956 | HMDB00510 | 12.544 | 1.10 | 0.035 | 1.25 | up |
|  | Cytidine | C00475 | HMDB00089 | 7.099 | 1.07 | < 0.001 | 4.98 | up |
|  | Choline | C00114 | HMDB00097 | 8.186 | 1.04 | 0.031 | 1.37 | up |
| PD vs. | 2-keto-D-Gluconic acid | C06473 | NA | 11.226 | 2.80 | < 0.001 | 3.72 | up |
| predialysis CKD-5 | Citraconic acid | C02226 | HMDB00634 | 1.647 | 2.75 | < 0.001 | 3.03 | up |
|  | S-Adenosylhomocysteine | C00021 | HMDB00939 | 11.718 | 2.05 | < 0.001 | 1.51 | up |
|  | D-Glucuronic acid | C00191 | HMDB00127 | 11.864 | 2.03 | < 0.001 | 1.63 | up |
|  | N2,N2-Dimethylguanosine | NA | HMDB04824 | 5.587 | 1.98 | < 0.001 | 2.47 | up |
|  | Kynurenic acid | C01717 | HMDB00715 | 5.749 | 1.90 | < 0.001 | 4.70 | up |
|  | L-2-Hydroxygluterate | C03196 | HMDB00694 | 12.047 | 1.78 | < 0.001 | 2.03 | up |
|  | Purine | C15587 | HMDB01366 | 3.575 | 1.73 | < 0.001 | 6.22 | up |
|  | L-histidinol | C00860 | HMDB03431 | 10.268 | 1.58 | < 0.001 | 2.00 | up |
|  | L-Glutamine | C00064 | HMDB00641 | 11.359 | 1.51 | 0.001 | 1.18 | down |
|  | N-Acetylglutamine | NA | HMDB06029 | 9.397 | 1.46 | 0.001 | 1.55 | up |
|  | Nicotinamide | C00153 | HMDB01406 | 1.371 | 1.42 | 0.004 | 1.37 | up |
|  | Creatinine | C00791 | HMDB00562 | 10.548 | 1.39 | < 0.001 | 1.09 | up |
|  | N-Acetyl-L-alanine | NA | HMDB00766 | 7.568 | 1.37 | 0.004 | 1.22 | up |
|  | Argininosuccinic acid | C03406 | HMDB00052 | 14.533 | 1.31 | 0.004 | 1.63 | up |
|  | Guanosine | C00387 | HMDB00133 | 7.757 | 1.29 | < 0.001 | 1.82 | up |
|  | Dihydroxy-acetone-phosphate | C00111 | HMDB01473 | 13.411 | 1.29 | 0.001 | 2.49 | up |
|  | 5'-Methylthioadenosine | C00170 | HMDB01173 | 2.213 | 1.28 | < 0.001 | 1.64 | up |
|  | L-Methionine sulfoxide | NA | HMDB02005 | 11.208 | 1.25 | 0.009 | 1.39 | down |
|  | L-Lactic acid | C01432 | HMDB01311 | 6.762 | 1.22 | 0.007 | 1.19 | up |
|  | L-Pipecolic acid | C00408 | HMDB00716 | 9.213 | 1.21 | 0.029 | 1.52 | up |
|  | Uridine | C00299 | HMDB00296 | 4.631 | 1.20 | 0.012 | 1.21 | up |
|  | L-Leucine | C00123 | HMDB00687 | 7.726 | 1.19 | 0.013 | 1.13 | down |
|  | Cytidine | C00475 | HMDB00089 | 7.099 | 1.14 | < 0.001 | 6.72 | up |
|  | Xanthurenic acid | C02470 | HMDB00881 | 6.477 | 1.10 | 0.019 | 1.45 | up |
|  | Taurine | C00245 | HMDB00251 | 8.793 | 1.07 | 0.027 | 1.26 | down |
|  | 4-Pyridoxic acid | C00847 | HMDB00017 | 1.002 | 1.02 | 0.004 | 2.13 | up |
| HD vs. PD | 2-keto-D-Gluconic acid | C06473 | NA | 11.226 | 3.42 | < 0.001 | 2.48 | down |
|  | Citraconic acid | C02226 | HMDB00634 | 1.647 | 2.98 | < 0.001 | 2.19 | down |
|  | L-Carnitine | C00318 | HMDB00062 | 10.763 | 2.57 | < 0.001 | 4.17 | up |
|  | Adenosine | C00212 | HMDB00050 | 4.989 | 1.83 | 0.003 | 1.28 | up |
|  | Creatinine | C00791 | HMDB00562 | 10.548 | 1.76 | 0.002 | 1.10 | down |
|  | Betaine | C00719 | HMDB00043 | 8.069 | 1.71 | 0.017 | 1.25 | down |
|  | S-Methyl-L-cysteine | NA | HMDB02108 | 8.723 | 1.68 | 0.003 | 1.43 | down |
|  | Nicotinamide | C00153 | HMDB01406 | 1.371 | 1.59 | 0.012 | 1.32 | down |
|  | L-2-Aminoadipic acid | C00956 | HMDB00510 | 12.544 | 1.46 | 0.014 | 1.28 | up |
|  | L-Pipecolic acid | C00408 | HMDB00716 | 9.213 | 1.46 | 0.005 | 1.54 | down |
|  | L-Glutamine | C00064 | HMDB00641 | 11.359 | 1.39 | 0.048 | 1.12 | up |
|  | Xanthosine | C01762 | HMDB00299 | 9.349 | 1.30 | 0.004 | 1.69 | down |
|  | Flavone | C15608 | HMDB03075 | 1.097 | 1.29 | 0.003 | 1.43 | up |
|  | L-Homocysteine | C00155 | HMDB00742 | 8.725 | 1.14 | 0.047 | 1.29 | down |
|  | Glycerophosphocholine | C00670 | HMDB00086 | 11.936 | 1.00 | 0.032 | 1.34 | down |

**Table S3. Metabolites involved in oxidative stress**

|  | **Metabolites** | **Oxidative stress** | **Fold change** | **Trend** |
| --- | --- | --- | --- | --- |
| HD vs. | Kynurenic acid [1,2] | anti-oxidant | 3.849 | up |
| predialysis CKD-5 | L-Carnitine [3] | anti-oxidant | 3.101 | up |
|  | S-Adenosylhomocysteine (SAH) [4,5] | pro-oxidant | 1.788 | up |
|  | S-Methyl-L-cysteine (SMC) [6,7] | anti-oxidant | 1.561 | down |
|  | Pantothenic acid [8] | anti-oxidant | 1.394 | up |
|  | FAD [9] | anti-oxidant | 1.188 | down |
|  | Creatine [10,11] | anti-oxidant | 1.377 | up |
|  | L-Homocysteine [12,13] | pro-oxidant | 1.413 | down |
|  | L-Serine [14] | anti-oxidant | 1.159 | down |
|  | Hypoxanthine [15,16] | pro-oxidant | 1.578 | up |
|  | Flavone [17] | pro-oxidant | 1.390 | up |
|  | Choline [18] | anti-oxidant | 1.365 | up |
| PD vs. | S-Adenosylhomocysteine (SAH) | pro-oxidant | 1.508 | up |
| predialysis CKD-5 | Kynurenic acid | anti-oxidant | 4.699 | up |
|  | L-Glutamine [19] | anti-oxidant | 1.178 | down |
|  | Nicotinamide [20] | pro-oxidant | 1.371 | up |
|  | L-Leucine [21] | anti-oxidant | 1.125 | down |
|  | Taurine [22] | anti-oxidant | 1.257 | down |
| HD vs. PD | L-Carnitine | anti-oxidant | 4.170 | up |
|  | Adenosine [23] | anti-oxidant | 1.277 | up |
|  | Betaine [24] | anti-oxidant | 1.251 | down |
|  | S-Methyl-L-cysteine (SMC) | anti-oxidant | 1.427 | down |
|  | Nicotinamide | pro-oxidant | 1.319 | down |
|  | L-Glutamine | anti-oxidant | 1.123 | up |
|  | Flavone | pro-oxidant | 1.431 | up |
|  | L-Homocysteine | pro-oxidant | 1.291 | down |

1. L.Z. Agudelo, D.M.S. Ferreira, I. Cervenka, et al, Kynurenic Acid and Gpr35 Regulate Adipose Tissue Energy Homeostasis and Inflammation. Cell Metab. 27 (2018) 378-392.

[R](https://www.ncbi.nlm.nih.gov/pubmed/?term=Lugo-Huitr%C3%B3n%20R%5BAuthor%5D&cauthor=true&cauthor_uid=21763768). Lugo-Huitrón,  [T](https://www.ncbi.nlm.nih.gov/pubmed/?term=Blanco-Ayala%20T%5BAuthor%5D&cauthor=true&cauthor_uid=21763768). Blanco-Ayala,  [P](https://www.ncbi.nlm.nih.gov/pubmed/?term=Ugalde-Mu%C3%B1iz%20P%5BAuthor%5D&cauthor=true&cauthor_uid=21763768). Ugalde-Muñiz, et al, On the antioxidant properties of kynurenic acid: free radical scavenging activity and inhibition of oxidative stress. [Neurotoxicol Teratol.](https://www.ncbi.nlm.nih.gov/pubmed/21763768) 33 (2011) 538-547.

1. E.L. Gill, S. Raman, R.A. Yost, et al, L-Carnitine Inhibits Lipopolysaccharide-Induced Nitric Oxide Production of SIM-A9 Microglia Cells. ACS Chem Neurosci. 9 (2018) 901-905.
2. Â. Zanatta, C. Cecatto, R.T. Ribeiro, et al, S-Adenosylmethionine promotes oxidative stress and decreases Na+, K+-ATPase activity in cerebral cortex supernatants of adolescent rats: implications for the pathogenesis of S-Adenosylhomocysteine hydrolase deficiency. Mol Neurobiol. (2017) doi: 10.1007/s12035-017-0804-z.
3. X. Luo, Y. Xiao, F. Song, et al, Increased plasma S-adenosyl-homocysteine levels induce the proliferation and migration of VSMCs through an oxidative stress-ERK1/2 pathway in apoE(-/-) mice. Cardiovasc Res. 95 (2012) 241-250.
4. R. Wassef, R. Haenold, A. Hansel, et al, Methionine sulfoxide reductase A and a dietary supplement S-methyl-L-cysteine prevent Parkinson's-like symptoms. J Neurosci. 27 (2007) 12808-12816.
5. S. Thomas, G.P. Senthilkumar, K. Sivaraman, et al, Effect of s-methyl-L-cysteine on oxidative stress, inflammation and insulin resistance in male wistar rats fed with high fructose diet. Iran J Med Sci. 40 (2015) 45-50.
6. X. Yi, J. Zhu, J. Zhang, et al, Investigation of the reverse effect of Danhong injection on doxorubicin-induced cardiotoxicity in H9c2 cells: Insight by LC-MS based non-targeted metabolomic analysis. J Pharm Biomed Anal. 152 (2018) 264-270.
7. K.R. Messner, J.A. Imlay, Mechanism of superoxide and hydrogen peroxide formation by fumarate reductase, succinate dehydrogenase, and aspartate oxidase. J Biol Chem. 277 (2002) 42563-42571.
8. S. Duarte-Silva, A. Neves-Carvalho, C. Soares-Cunha, et al, Neuroprotective effects of creatine in the CMVMJD135 mouse model of spinocerebellar ataxia type 3. Mov Disord. (2018) doi: 10.1002/mds.27292.
9. R. Deminice, F.T. Rosa, G.S. Franco, et al, Effects of creatine supplementation on oxidative stress and inflammatory markers afterrepeated-sprint exercise in humans. Nutrition. 29 (2013) 1127-1132.
10. Z.P.You, Y.Z. Zhang, Y.L. Zhang, et al, Homocysteine induces oxidative stress to damage trabecular meshwork cells. Exp Ther Med. 15 (2018) 4379-4385.
11. C.H. Ma, Y.C. Chiua, C.H. Wu, et al, Homocysteine causes dysfunction of chondrocytes and oxidative stress through repression of SIRT1/AMPK pathway: A possible link between hyperhomocysteinemia and osteoarthritis. Redox Biol. 15 (2018) 504-512.
12. M.N. Maralani, A. Movahedian, ShH. Javanmard, Antioxidant and cytoprotective effects of L-Serine on human endothelial cells. Res Pharm Sci. 7 (2012) 209-215.
13. A.F. Rodrigues, R. Roecker, G.M. Junges, et al, Hypoxanthine induces oxidative stress in kidney of rats: protective effect of vitamins E plus C and allopurinol. Cell Biochem Funct. 32 (2014) 387-394.
14. Y.J. Kim, H.M. Ryu, J.Y. Choi, et al, Hypoxanthine causes endothelial dysfunction through oxidative stress-induced apoptosis. Biochem Biophys Res Commun. 482 (2017) 821-827.
15. U. Wenzel, A. Nickel, H. Daniel, Increased mitochondrial palmitoylcarnitine/carnitine countertransport by flavone causes oxidative stress and apoptosis in colon cancer cells. Cell Mol Life Sci. 62 (2005) 3100-3105.
16. A.K. Mehta, N. Arora, S.N. Gaur, et al, Choline supplementation reduces oxidative stress in mouse model of allergic airway disease. Eur J Clin Invest. 39 (2009) 934-941.
17. O.B. Ocheja, J.O. Ayo, T. Aluwong, et al, Effects of L-glutamine on rectal temperature and some markers of oxidative stress in Red Sokoto goats during the hot-dry season. Trop Anim Health Prod. 49 (2017) 1273-1280.
18. Y. Feng, Y. Wang, C. Jiang, et al, Nicotinamide induces mitochondrial-mediated apoptosis through oxidative stress in human cervical cancer HeLa cells. Life Sci. 181 (2017) 62-69.
19. L.F. Stoppiglia, T.A. Nogueira, A.R. Leite, et al, Protective effect of D-glucose, L-leucine and fetal calf serum against oxidative stress in neonatal pancreatic islets. Biochim Biophys Acta. 1588 (2002) 113-118.
20. K.H. Reeta, D. Singh, Y.K. Gupta, Chronic treatment with taurine after intracerebroventricular streptozotocin injection improves cognitive dysfunction in rats by modulating oxidative stress, cholinergic functions and neuroinflammation. Neurochem Int. 108 (2017) 146-156.
21. V. Ramkumar, D.M. Hallam, Z. Nie, Adenosine, oxidative stress and cytoprotection. Jpn J Pharmacol. 86 (2001) 265-274.
22. H. Hagar, W. Al Malki, Betaine supplementation protects against renal injury induced by cadmium intoxication in rats: role of oxidative stress and caspase-3. Environ Toxicol Pharmacol. 37 (2014) 803-811.

**Figure S1.** **Typical total ion chromatograms of the 3 representative plasma samples for predialysis CKD-5, HD, and PD groups**


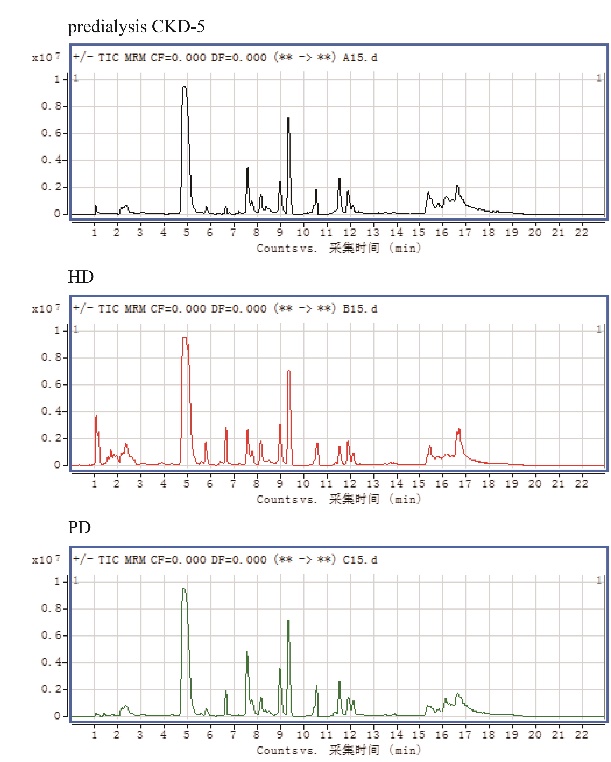

Supplement: Supplementary file 1 [file Data_Sheet_1.docx]
